# Effect of Virtual Reality Glasses and Melody on Cortisol and Adrenocorticotropic Hormone Levels in Patients With Knee Replacement Surgery Under Combined Spinal Epidural Anaesthesia

**DOI:** 10.7759/cureus.63017

**Published:** 2024-06-24

**Authors:** Nisha Singh, Shashank kumar Kanaujia, Manish K Singh, Nidhi Shukla, Ahsan k Siddiqui, Monica Kohli

**Affiliations:** 1 Anesthesia and Critical Care, King George's Medical University, Lucknow, IND; 2 Anaesthesiology, Integral Institute of Medical Sciences and Research, Lucknow, IND

**Keywords:** acth, noise cancelling headphones, virtual reality, music, knee replacement, cortisol

## Abstract

Background: With advanced virtual reality (VR) technology, its usage in health care is creating an impact on patient outcomes. Patients undergoing knee replacement surgery are already anxious due to the surgery, anaesthesia, and unfamiliar environment of the operation theatre. In addition to that, the unpleasant noise of tools makes it worse. Peri-operative anxiety correlates with increased anaesthesia requirements and prolonged recovery. It causes the release of stress hormones such as cortisol, adrenaline, and norepinephrine, which can lead to difficult intravascular access due to vasoconstriction and heightened cardiovascular responses. Studies on music therapy have shown a reduction in cortisol levels, contributing to anxiety alleviation. VR glasses create immersive environments to distract patients from various stress factors. Investigating the use of VR/music on serum cortisol and adrenocorticotropic hormone (ACTH) levels in knee replacement surgery can improve peri-operative care, improving patient outcomes.

Aim: The study was done to investigate the impact of virtual reality glasses and music therapy on serum cortisol and ACTH levels in patients undergoing knee replacement surgery under combined spinal epidural anaesthesia.

Methods: In this prospective randomised control, single-centric study, patients of either sex, aged between 18 and 65 years, undergoing knee replacement surgery under combined spinal and epidural (CSE) anaesthesia, were included. The primary objective was to compare serum cortisol and ACTH levels, while the secondary objective was to compare the State-Trait Anxiety Inventory for State Anxiety (STAI-SA) score and Patient Satisfaction Score (PSS) in the peri-operative period. A total of 100 patients were assessed for eligibility, and 66 patients met the inclusion and exclusion criteria and were finally randomised and equally assigned to group M-VR (music-virtual reality) and group C (control). Three blood samples were collected for serum cortisol and serum ACTH levels one hour before surgery (T1), one hour after skin incision (T2), and two hours after the completion of surgery (T3). STAI-SA was measured one hour before surgery (T1) and two hours after the completion of surgery (T2), while PSS was recorded two hours after the completion of surgery. Hemodynamic parameters were noted during the entire peri-operative period.

Results: The demographic and anthropometric parameters were comparable in both groups. Hemodynamic parameters (heart rate [HR], mean arterial pressure [MAP]) were found to be comparable in the pre-operative period, while significant differences (p > 0.05) were noted after 30 minutes of surgery and continued till the end of surgery. Serum cortisol and serum ACTH levels were comparable in the pre-operative period but showed significantly lower variations in group M-VR in comparison to group C in the intra-operative period. PSS was significantly higher in group M-VR in comparison to group C.

Conclusion: This study substantiates the role of virtual reality and music therapy (VR/music) on anxiety reduction, improved satisfaction scores, and lesser ACTH/cortisol level variations in knee replacement surgery. It further emphasises larger randomised controlled studies in various other surgical populations, along with long-term follow-up and outcome assessment.

## Introduction

Peri-operative anxiety is common in surgical settings, especially knee replacement surgery done under combined spinal and epidural (CSE); patients are aware of operating room noise and orthopaedic activities like oscillating saws and hammers, potentially adding stress levels and requiring additional sedatives to keep patients calm and composed. Non-pharmacological interventions like virtual reality (VR) glasses and music therapy have emerged as tools to deal with it [1-7].

Stress triggers adrenocorticotropic hormone (ACTH)-induced cortisol release with a normal diurnal rhythm, which gets altered in a chronic stress state and renders the stress response ineffective [8-10]. The existing literature has shown that listening to pleasant music leads to a reduction in cortisol levels [11,12]. Music therapy, which induces relaxation and reduces anxiety by engaging attention channels in the brain with soothing auditory stimuli, has been found to alleviate anxiety in awake surgical procedures and improve pain management and patient satisfaction even under general anaesthesia [13,14]. It was found that patient-preferred music is particularly effective, offering a cost-effective and accessible tool to mitigate the stress response associated with surgery [15,16]. Nowadays, VR glasses have emerged as a promising tool to alleviate peri-operative anxiety by creating immersive environments that distract patients from stressful situations [17,18]. However, the role of virtual reality glasses and melody on peri-operative stress-led ACTH/cortisol level variations in knee replacement surgeries is an under-explored area of research.

## Materials and methods

This prospective randomised controlled study conducted at the Department of Anaesthesiology, in collaboration with the Department of Orthopaedics, King George’s Medical University, Lucknow, 2073/ethics/2023, and clinical trial registry India CTRI/2023/06/053355, aimed to investigate the impact of VR glasses and music on cortisol and ACTH levels during knee replacement surgery for a period of one year. The sample size was calculated based on a study done by Leardi et al., where the primary objective was to measure changes in serum cortisol levels using music. The changes in the cortisol level in the new age music group (14.21) and listening to a choice of music (8.63) in stress response to daycare surgery were 5.58, and the population variance (*σ*^2^) was 6.69.

The sample size (*n*) = 2 (*Z*_*α*/2_ + *Z* _[1-*β*]_)^2^ × *σ*^2^/(*μ*_1_−*μ*_2_)^2^, assuming 0.05 level significance (*Z*_*α*/2_ = 1.96) and 90% power (*Z*_[1−*β*]_)=1.28) was 33 in each group. In this study, we will enroll 33 patients in each group

*n *= 2 (*Z*_*α*/2_ + *Z*_[1-*β*]_)^2^ × *σ*^2^/(*μ*_1_−*μ*_2_)^2^

*n* = 2 (1.96 + 1.28)^2^ × 6.962/(14.21−8.63)^2^

*n* = 32.65

**Figure 1:**
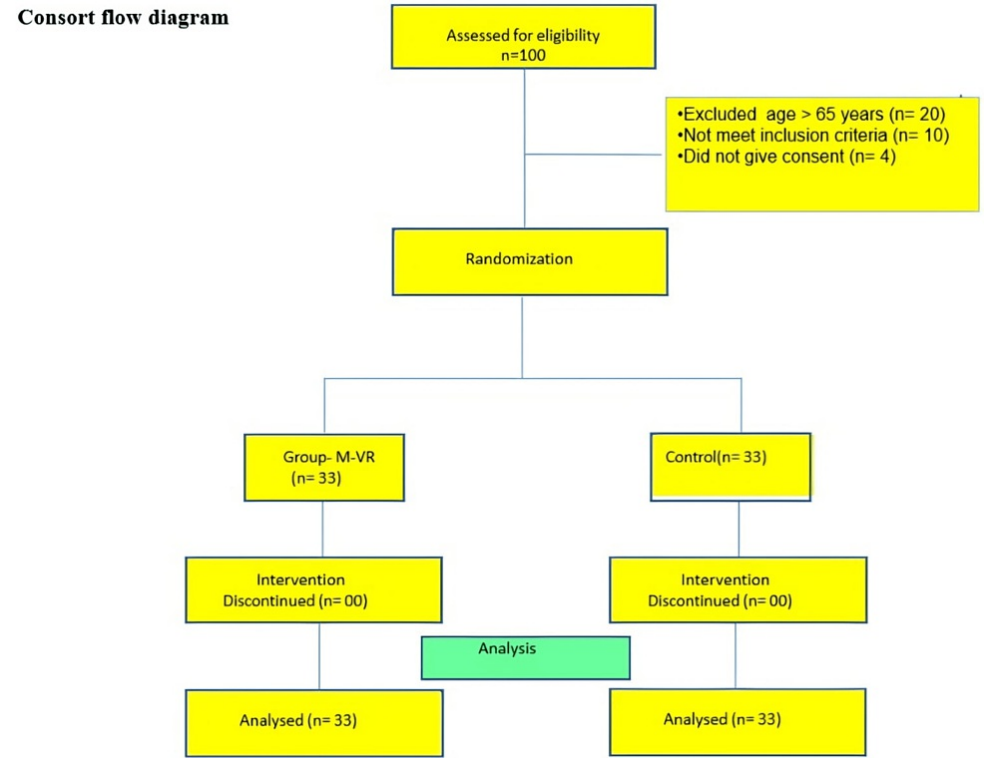
Consort diagram.

All patients with ASA (American Society of Anesthesiologists) grades I, II, and III aged between 18 and 65 years scheduled for unilateral knee replacement surgery under combined spinal epidural anaesthesia were enrolled. Patients with hearing and visual impairment, a deranged coagulation profile, anatomical defects or active infections of the spine, neurological or psychiatric disorders, chronic pain syndrome, or an anticipated duration of surgery of more than three hours were excluded. In total, 100 patients were enrolled in the study. Out of these, 20 patients were excluded who were above 65 years of age, 10 patients did not meet inclusion criteria, and 4 patients did not give consent for our intervention (Figure 1).

**Figure 2:**
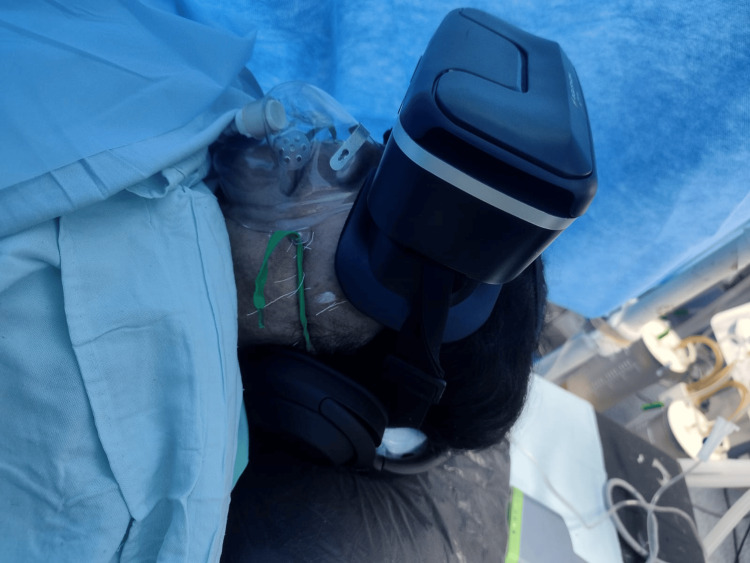
Patient with virtual reality device IRUSU MONSTER VR (@ 2014, Hyderabad, India) and noise cancelling headphone SONY WH-1000XM4 (Japan).

After obtaining written and informed consent, 33 participants were randomly assigned to groups C (control) and M-VR (music-virtual reality) using computer-generated random numbers. Approximately one hour before the start of surgery, participants completed the State-Trait Anxiety Inventory for State Anxiety (STAI-SA) questionnaire, and the first blood sample (T1) was collected to establish the baseline levels of cortisol and ACTH. Participants in group M-VR were provided with an IRUSU MONSTER VR headset and noise-cancelling headphones from Sony WH-1000XM4 (Figure 2) and were asked to watch a video of their choice, while in group C, the surgery commenced without any additional steps. The video was temporarily paused, and the patient was then moved to the operating room. An intravenous (IV) access was obtained, and the patient was co-loaded with 500-1000 ml of Ringer lactate solution. Baseline heart rate (HR), non-invasive blood pressure (NIBP), oxygen saturation (SpO_2_), and ECG were recorded. The patient was made to sit up on the operation theatre table and, with the help of aseptic preparation combined with spinal epidural anaesthesia, was given at L3-L4 level by bupivacaine heavy 0.5% (0.25 mg/kg) and fentanyl (25 μg), followed by sedation with 0.02 mg/kg midazolam in both groups. After the adequate sensory and motor block, patients in the group M-VR were provided virtual reality glasses along with noise-cancelling headphones and asked to watch a video of their choice. Following this, a bolus of bupivacaine (0.25%) 8 ml was administered via epidural catheter during the intra-operative period at the end of surgery. In case any patient experiences discomfort, such as nausea, vomiting, etc., during the surgery, the video will be stopped, and that patient will be excluded from the study.

Post-operatively, after two hours, only a 0.125% bupivacaine and a 2 µg/ml fentanyl infusion were continued in both groups at 5 ml/hour, according to vitals. Vital parameters were assessed pre-operatively and intra-operatively at 0 minutes, 5 minutes, 15 minutes, 30 minutes, 45 minutes, 1 hour, 1 hour, 15 minutes, and every 15 minutes till the end of surgery. The second blood sample (T2) and a third blood sample (T3) were drawn after one hour of skin incision and two hours after completion of surgery (post-operative period), respectively.

**Figure 3:**
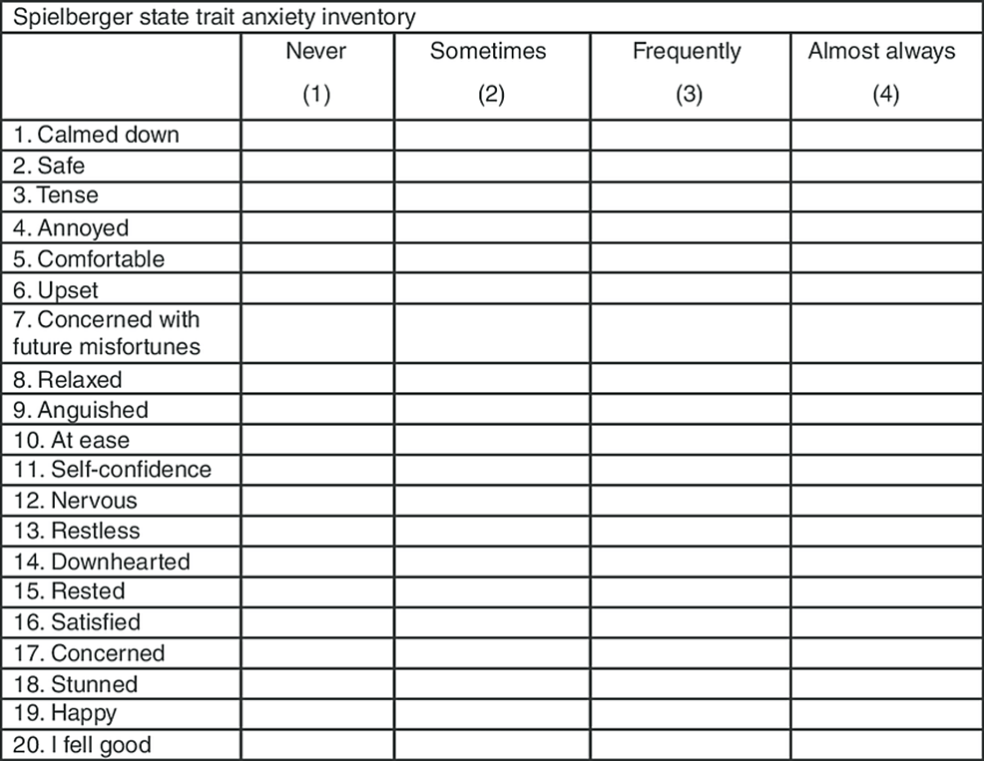
State-Trait Anxiety Inventory (STAI) scoring.

The primary objective of the study was to compare the role of virtual reality glasses and melody on cortisol and ACTH level variations during knee replacement surgery under CSE. The secondary objectives were to compare the anxiety state using the STAI-SA (Figure 3) and measure the patient satisfaction score (PSS) (Table 1).

**Table 1:** Comparison of level of patient satisfaction. Patient satisfaction was assessed using a satisfaction scale, where 1 represents "very satisfied," 2 represents "satisfied," 3 represents "dissatisfied," and 4 represents "very dissatisfied.

Level of patient satisfaction score	
1	Very satisfied
2	Satisfied
3	Dissatisfied
4	Very dissatisfied

The statistical analysis was done using SPSS (Statistical Package for Social Sciences) version 21.0 statistical analysis software (IBM Corp., Armonk, NY). The values were represented in numbers (%) and mean ± SD. The chi-square (χ2) test was used to compare the proportional differences. The student's ‘t’ test has been used to compare the continuous data of two groups, represented as mean ± SD. Intra-group change has been assessed using the paired ‘t’ test.

## Results

In both groups, there are statistically significant changes present, as shown in Table 2. The demographic profile and anthropometric measurements were comparable between the two groups (Tables 3-4). The heart rates of the two study groups were comparable pre-operatively as well as during the initial 30 minutes of surgery. At 45 minutes, 1 hour, and 1 hour 15 minutes intra-operatively and at the end of the surgery, the heart rate of the control group (group C) was statistically significant and higher as compared to group M-VR (Table 5).

**Table 2:** Intragroup change in baseline MAP at different time intervals. MAP: mean arterial pressure. *Intra-group MAP has been compared. In group M-VR, a decline in baseline heart rate was observed at all the periods of observation except at 0 minutes. The change in baseline heart rate was statistically significant at all the periods of observation except for the 0-minute and 5-minute intra-operative periods. The maximum change in baseline heart rate was observed at 1 hour 15 minutes (13.35%) and at one hour (12.83%), while the minimum change in baseline heart rate was observed at five minutes (0.94%), followed by a 0-minute intra-operative period (3.41%). In group C, heart rate was found to be below heart rate at all the periods of observation except at the 0-minute and 5-minute intra-operative periods. The maximum percentage change in baseline heart rate was observed at 0 minutes (4.21%), followed by 30 minutes and 45 minutes (3.59%), while a minimum change was observed at the end of surgery (0.72%), followed by a five-minute intra-operative period (1.34%). A statistically significant change in baseline heart rate was observed at 0-minute, 30-minute, 45-minute, and 1-hour 15-minute intra-operative periods.

	Group M-VR					Control group C				
	Mean diff	SD	% Ch	‘t’	‘p’	Mean diff	SD	% Ch	‘t’	‘p’
0 min	−4.30	7.27	−4.61	−3.398	0.002	1.22	4.51	1.36	1.557	0.129
5 min	−13.12	6.87	−14.06	−10.965	<0.001	−3.23	6.83	−3.59	−2.717	0.011^*^
15 min	−14.37	8.43	−15.41	−9.792	<0.001	−3.29	8.38	−3.66	−2.258	0.031^*^
30 min	−13.93	7.58	−14.93	−10.550	<0.001	−5.08	6.90	−5.64	−4.233	<0.001^*^
45 min	−11.58	6.64	−12.41	−10.010	<0.001	−4.28	6.86	−4.76	−3.586	0.001^*^
1 hour	−11.26	8.73	−12.07	−7.409	<0.001	−2.34	7.16	−2.60	−1.880	0.069
1 hour 15 min	−10.02	8.67	−10.74	−6.639	<0.001	−0.35	7.95	−0.39	-0.255	0.800
SurgEnd	−8.49	8.74	−9.11	−5.581	<0.001	3.54	9.17	3.93	2.214	0.034^*^

**Table 3:** Comparison of demographic profile of two study groups.

SN	Characteristics	Total (N=66)	Group M-VR (n=33)		Group C (n=33)		Statistical significance	
			Mean	SD	Mean	SD	‘t’	‘p’
1	Mean age ± SD	53.42±12.89	53.67	±14.28	53.18	±11.54	0.152	0.880
	Range in years	(18−75)	(18−75)		(30−75)			
2	Gender		No.	%	No.	%	c²	‘p’
	Female	32 (48.5%)	13	39.4	19	57.6	2.184	0.139
	Male	34 (51.5%)	20	60.6	14	42.4		

**Table 4:** Comparison of anthropometric parameters of two study groups.

SN	Characteristics	Group M-VR (n=33)		Group C (n=33)		Statistical significance	
		Mean	SD	Mean	SD	‘t’	‘p’
1	Weight (kg)	65.97	7.42	63.24	8.15	1.422	0.160
2	Height (cm)	158.58	4.10	159.88	5.98	−1.033	0.306
3	BMI (kg/m²)	26.28	3.12	24.77	3.20	1.930	0.058
4	BMI (category)	No.	%	No.	%	c²	‘p’
	Normal (18.5–24.9)	11	33.3	16	48.5	2.059	0.357
	Overweight (25–29.9)	16	48.5	14	42.4		
	Obese (≥30)	6	18.2	3	9.1		

**Table 5:** Comparison of heart rate of two study groups at different time intervals. M-VR: music-virtual reality; PRE-OP: preoperatively. *At 45 minutes, 1 hour, 1 hour 15 minutes, and the end of the surgery, the heart rate is lower in the M-VR group as compared to control group C, with a p-value of <0.05, which is statistically significant.

SN	Time interval	Group M-VR (n=33)		Control group C (n=33)		Statistical significance	
		Mean	SD	Mean	SD	‘t’	‘p’
1	Preop	93.30	17.70	92.79	17.18	0.120	0.905
2	0 min	96.48	23.12	96.70	17.63	−0.042	0.967
3	5 min	92.42	17.78	94.03	15.49	−0.391	0.697
4	15 min	86.67	17.14	91.52	14.18	−1.252	0.215
5	30 min	83.36	16.61	89.45	13.30	−1.644	0.105
6	45 min	82.18	14.31	89.45	13.29	−2.140	0.036^*^
7	1 hour	81.33	15.40	89.97	14.49	−2.346	0.022^*^
8	1 hour 15 min	80.85	15.45	89.58	14.15	−2.393	0.020^*^
9	End of Surg	82.88	14.98	92.12	13.06	−2.672	0.010^*^

The mean arterial pressure of group M-VR was found to be significantly lower than its baseline levels at all the intra-operative periods of observation. The minimum percentage change in baseline MAP was observed at 0 minutes (4.61%), followed by at the end of surgery (9.11%), while the maximum change was observed at 15 minutes (15.41%), followed by 30 minutes (14.93%). In the control group, MAP was found to be significantly higher than its baseline value at 0 minutes and at the end of surgery. During the rest of the intra-operative period, MAP was found to be lower than its baseline values. A significant change in baseline MAP was observed at all the periods of observation except at 0 minutes, 1 hour, and 1 hour 15 minutes (Table 6).

**Table 6:** Comparison of mean arterial pressure of two study groups at different time intervals. MAP: mean arterial pressure. *The mean arterial pressure of groups M-VR and C was comparable at baseline, at 0 minutes, and at 45 minutes. During the rest of the periods of observation, the mean arterial pressure of group M-VR was lower than that of group C, which was statistically significant.

SN	Time interval	Group M-VR (n=33)		Group C (n=33)		Statistical significance	
		Mean	SD	Mean	SD	‘t’	‘p’
1	Preop	93.29	8.29	90.05	11.79	1.293	0.201
2	0 min	88.99	9.77	91.27	11.81	−0.856	0.395
3	5 min	80.17	8.57	86.82	12.10	−2.575	0.012^*^
4	15 min	78.92	8.89	86.76	12.61	−2.918	0.005^*^
5	30 min	79.36	10.05	84.97	10.08	−2.262	0.027^*^
6	45 min	81.72	8.88	85.77	9.05	−1.836	0.071
7	1 hour	82.03	9.05	87.71	9.29	−2.514	0.014^*^
8	1 hour 15 min	83.27	8.23	89.70	9.38	−2.958	0.004^*^
9	End of Surg	84.80	8.62	93.59	8.85	−4.086	<0.001^*^

Serum cortisol levels of group M-VR and controls were comparable at T1 and T3, while lower S. cortisol was observed in group M-VR as compared to controls at T2, which was statistically significant. The serum ACTH levels of group M-VR and controls were comparable at T1, while lower S. ACTH in group M-VR as compared to controls was observed at T2 and T3, which was statistically significant (Table 7).

**Table 7:** Comparison of STAI Score, S. cortisol, and S. ACTH of two study groups at different time intervals. *S. ACTH (serum adrenocorticotropic hormone), T1: 1 hour before surgery, T2: 1 hour after skin incision, T3: 2-hour post-operative period. In STAI (State-Trait Anxiety Inventory scoring), T1: 1 hour before surgery scoring, T2: 2-hour post-op scoring, there is no T3. At T1, the STAI score of group M-VR was higher as compared to group C (53.73±7.27 vs. 51.88±5.69), but this difference was not found to be significant statistically. At the T2 STAI score of the group, M-VR was lower as compared to group C (39.09±4.13 vs. 63.15±7.16), which was statistically significant.

SN	Time interval	Group M-VR (n=33)		Control Group C (n=33)		Statistical significance	
		Mean	SD	Mean	SD	‘t’	‘p’
1	STAI score						
	T1	53.73	7.27	51.88	5.69	1.150	0.254
	T2	39.09	4.13	63.15	7.16	−16.719	<0.001^*^
2	S. cortisol (mcg/dl)						
	T1	28.54	17.96	28.33	15.43	0.051	0.959
	T2	16.65	19.57	39.15	20.15	−4.600	<0.001^*^
	T3	23.02	22.47	40.50	18.88	−3.423	0.001^*^
3	S. ACTH (pg/ml)						
	T1	54.02	24.43	64.94	20.23	−1.976	0.053
	T2	33.96	19.18	81.84	19.90	−9.953	<0.001^*^
	T3	36.87	20.93	77.59	16.96	−8.685	<0.001^*^

The patient satisfaction level of the majority of group M-VR patients was level 1-2 (97.0%), while the majority of group C had a patient satisfaction level of levels 3-4 (81.8%). None of the group C patients had a level 1 satisfaction level, while none of the group M-VR patients had a level 4 satisfaction level. This difference was found to be significant statistically (p<0.001) (Table 8).

**Table 8:** Comparison of level of patient satisfaction. Patient satisfaction was assessed using a satisfaction scale, where 1 represents "very satisfied," 2 represents "satisfied," 3 represents "dissatisfied," and 4 represents "very dissatisfied.

Level of patient satisfaction	Total (N=66)	Group M-VR (n=33)		Group C (n=33)	
		No.	%	No.	%
Level 1	15	15	45.5	0	0.0
Level 2	23	17	51.5	6	18.2
Level 3	21	1	3.0	20	60.6
Level 4	7	0	0.0	7	21.2

## Discussion

Surgery often induces anxiety in patients, adversely impacting both their psychological well-being and post-operative recovery. Moreover, patients’s awareness of regional anaesthesia cases increases their anxiety level. Numerous research studies have investigated the positive effects of music therapy on enhancing peri-operative care quality [19]. Similarly, Uedo et al. also observed decreased cortisol levels in the music group as compared to the control group [20]. Mosso et al. discovered a significant reduction in cortisol concentration following VR usage [21]. The process of familiarisation and anticipation, known as habituation, could lead to the effective alleviation of fear responses [22]. Additionally, patients may discern the distinction between imagined fear and reality within the VR environment [22]. Second, VR can engage patients with audio-visual content, diverting their attention away from preoperative anxiety [23]. Although the superiority of VR over other non-pharmacological interventions regarding its impact on preoperative anxiety remains uncertain, a recent systemic review suggested that VR might be more efficacious than other non-pharmacological methods [24,25]. While cortisol is crucial for adapting to environmental challenges, patients commonly express a strong aversion to the heightened pre- and intra-operative stress, worry, and anxiety. Consequently, our finding of the least fluctuation of cortisol levels in the M-VR group is corroborative as a favourable outcome and as evidence of lesser stress and anxiety in this group. The potential medical consequences of varying cortisol levels during surgery are yet to be clearly defined [26]. Additionally, the serum ACTH levels of groups M-VR and C were comparable at baseline, and significantly lower serum ACTH in group M-VR compared to controls during intra-operative care was observed in our study (p < 0.001). In our study, participant anxiety levels were assessed using the STAI, comprising the STAI-SA. The STAI score of group M-VR was significantly lower as compared to group C. Our results were in alignment with the study by Baran et al.; the scores on the STAI scale were significantly diminished in the video group compared to the control group (p = 0.000; p < 0.005) [26]. Maeyama et al. observed the positive effects of music on anxiety, noting a reduction in the Bispectral Index (BIS) value during spinal anaesthesia (p < 0.01). Pre- and post-operatively, STAI-TA scores were obtained using STAI-TA. The findings revealed a significantly lower STAI-TA in the music group compared to the control group [27]. We also found in our study that group M-VR exhibited lower anxiety scores (STAI), cortisol, and ACTH levels, particularly notable during the intra-operative period. These findings suggest that M-VR intervention may contribute to improved physiological responses and reduced anxiety during surgery compared to conventional methods. About 97% of patients in group M-VR were found to have a level 1-2 satisfaction score, while merely 18.2% of patients in group C had a similar level of satisfaction; these findings were very similar to the results of Mumm et al. [28]. We did not find any complications of using VR, like eye strain, headaches, nausea, and vomiting, among any patients in group M-VR. The limitations of our study were a similar subset of the surgical population, a small sample size, and the absence of post-operative pain assessment.

## Conclusions

This study substantiates the role of virtual reality and music therapy (VR/music) on anxiety reduction, improved satisfaction scores, and lesser ACTH/cortisol level variations in knee replacement surgery. It further emphasises larger randomised controlled studies in various other surgical populations, along with long-term follow-up and outcome assessment.

## References

[1] Kukreja P, Talbott K, MacBeth L (2020). Effects of music therapy during total knee arthroplasty under spinal anesthesia: a prospective randomized controlled study. Cureus.

[2] Chen SB, Hu H, Gao YS, He HY, Jin DX, Zhang CQ (2015). Prevalence of clinical anxiety, clinical depression and associated risk factors in chinese young and middle-aged patients with osteonecrosis of the femoral head. PLoS One.

[3] Weissman C (1990). The metabolic response to stress: an overview and update. Anesthesiology.

[4] Mulugeta H, Ayana M, Sintayehu M, Dessie G, Zewdu T (2018). Preoperative anxiety and associated factors among adult surgical patients in Debre Markos and Felege Hiwot referral hospitals, Northwest Ethiopia. BMC Anesthesiol.

[5] Maranets I, Kain ZN (1999). Preoperative anxiety and intraoperative anesthetic requirements. Anesth Analg.

[6] Ali A, Altun D, Oguz BH, Ilhan M, Demircan F, Koltka K (2014). The effect of preoperative anxiety on postoperative analgesia and anesthesia recovery in patients undergoing laparascopic cholecystectomy. J Anesth.

[7] Bansal P, Kharod U, Patel P, Sanwatsarkar S, Patel H, Kamat H (2010). The effect of music therapy on sedative requirements and haemodynamic parameters in patients under spinal anaesthesia: a prospective study. J Clin Diagn Res.

[8] Simpson JP, Hamer AJ (2017). How noisy are total knee and hip replacements?. J Perioper Pract.

[9] Ehlert U, Gaab J, Heinrichs M (2001). Psychoneuroendocrinological contributions tothe etiology of depression, posttraumatic stress disorder, and stress-related bodily disorders: the role of the hypothalamus-pituitary-adrenal axis. Biol Psychol.

[10] Heim C, Ehlert U, Hellhammer DH (2000). The potential role of hypocortisolism in the pathophysiology of stress-related bodily disorders. Psychoneuroendocrinology.

[11] Tsigos C, Chrousos GP (2002). Hypothalamic-pituitary-adrenal axis, neuroendocrine factors and stress. J Psychosom Res.

[12] McKinney CH, Antoni MH, Kumar M, Tims FC, McCabe PM (1997). Effects of guided imagery and music (GIM) therapy on mood and cortisol in healthy adults. Health Psychol.

[13] VanderArk SD, Ely D (1992). Biochemical and galvanic skin responses to music stimuli by college students in biology and music. Percept Mot Skills.

[14] Kahloul M, Mhamdi S, Nakhli MS, Sfeyhi AN, Azzaza M, Chaouch A, Naija W (2017). Effects of music therapy under general anesthesia in patients undergoing abdominal surgery. Libyan J Med.

[15] Wan X, Wang W, Liu J, Tong T (2014). Estimating the sample mean and standard deviation from the sample size, median, range and/or interquartile range. BMC Med Res Methodol.

[16] Labbé E, Schmidt N, Babin J, Pharr M (2007). Coping with stress: the effectiveness of different types of music. Appl Psychophysiol Biofeedback.

[17] Hole J, Hirsch M, Ball E, Meads C (2015). Music as an aid for postoperative recovery in adults: a systematic review and meta-analysis. Lancet.

[18] Székely G, Satava RM (1999). Virtual reality in medicine. Interview by Judy Jones. BMJ.

[19] Ganry L, Hersant B, Sidahmed-Mezi M, Dhonneur G, Meningaud JP (2018). Using virtual reality to control preoperative anxiety in ambulatory surgery patients: a pilot study in maxillofacial and plastic surgery. J Stomatol Oral Maxillofac Surg.

[20] Uedo N, Ishikawa H, Morimoto K (2004). Reduction in salivary cortisol level by music therapy during colonoscopic examination. Hepatogastroenterology.

[21] Mosso JL, Gorini A, De La Cerda G (2009). Virtual reality on mobile phones to reduce anxiety in outpatient surgery. Stud Health Technol Inform.

[22] Sjöling M, Nordahl G, Olofsson N, Asplund K (2003). The impact of preoperative information on state anxiety, postoperative pain and satisfaction with pain management. Patient Educ Couns.

[23] Jlala HA, French JL, Foxall GL, Hardman JG, Bedforth NM (2010). Effect of preoperative multimedia information on perioperative anxiety in patients undergoing procedures under regional anaesthesia. Br J Anaesth.

[24] Riva G (2003). Virtual environments in clinical psychology. Psychother Theory Res Pract Train.

[25] Emmelkamp PM, Meyerbröker K (2021). Virtual reality therapy in mental health. Annu Rev Clin Psychol.

[26] Baran O, Mordeniz C, Arar MC, Günkaya M (2020). The effect of video information on preoperative anxiety levels in patients undergoing total knee replacement. J Acad Res Med.

[27] Maeyama A, Kodaka M, Miyao H (2009). [Effect of the music-therapy under spinal anesthesia]. Masui.

[28] Mumm JN, Eismann L, Rodler S (2021). Listening to music during outpatient cystoscopy reduces pain and anxiety and increases satisfaction: results from a prospective randomised study Jan-Niclas mum. Urol Int.

